# An empirical assessment of the factors influencing acceptance of COVID-19 vaccine uptake between Kenyan and Hungarian residing populations: A cross-sectional study

**DOI:** 10.1038/s41598-022-26824-5

**Published:** 2022-12-23

**Authors:** John M. Macharia, Grace W. Gakenye, Nóra Rozmann, David Onchonga, Ruth W. Mwangi, Zsolt Kaposztas, John M. Mathenge, Dorina Pusztai, Marton Pinter, Miklos Sugar, Bence L. Raposa

**Affiliations:** 1 Doctoral School of Health Sciences, Faculty of Health Science, University of Pecs, Vorosmarty Mihaly Str. 4, Pecs, 7621 Hungary; 2 Faculty of Business and Economics, University of Pecs, Pecs, Hungary; 3 School of Medicine, University of Limerick, Limerick, Ireland; 4 Faculty of Science, Department of Biological Sciences, Egerton University, Nakuru, Kenya; 5 Doctoral School of Horticultural Sciences, Institute of Vegetables and Mushroom Growing, Hungarian University of Agriculture and Life Sciences, Budapest, Hungary; 6 Faculty of Health Science, University of Pẻcs, Pẻcs, Hungary; 7 School of Agriculture and Enterprise Development, Kenyatta University, Nairobi, Kenya

**Keywords:** Health care, Health occupations

## Abstract

The development of effective, safe, and acceptable vaccines is a long process. COVID-19 vaccine hesitancy continues to elicit mixed reactions among different quarters despite numerous evidence of their effectiveness. This study aimed to determine the availability and acceptance rates of SARS-CoV-2 vaccines, among Kenyan and Hungarian residing populations and the underlying reasons contributing to the hesitancy of uptake. A non-probability, snowball sampling design was employed, and a survey questionnaire tool link was expeditiously disseminated. Data were carefully analyzed descriptively. Demographic variables, COVID-19 awareness, possible exposure, reasons associated with hesitancy in taking up a vaccine, choice of a vaccine, and availability of vaccines among other important variables were tested to explore their associations with vaccine acceptance rates between the two distinct countries. A total of 1960 participants were successfully enrolled in the research study, while 67 participants were excluded based on the inclusion criterion set. There was, however, no significant difference in COVID-19 public awareness between the Kenyan and Hungarian-residing participants, *p* = 0.300. Of the respondents, 62.4% were willing and ready to receive vaccines against COVID-19 disease. There was a significant difference (*p* = 0.014) between the Kenyan and Hungarian-residing respondents concerning vaccine uptake and acceptance rates. The vaccine acceptance rates in Hungary were higher than in Kenya, with mean = 0.27, SD = 0.446, S. E = 0.045 for the Hungarian population sample and mean = 0.40, SD = 0.492, S. E = 0.026, for the Kenyan sample respectively. Concerning gender and vaccine acceptance, there was a notable significant difference between males and females, *p* = 0.001, where the mean for males and females were 0.29 and 0.46 respectively. Acceptance rates among males were higher than among females. The functions of One-Way ANOVA and Chi-square were used to establish any significant differences and associations between means and variables respectively. Concerns regarding the safety, efficacy, and accuracy of information about the developed vaccines are significant factors that must be promptly addressed, to arrest crises revolving around COVID-19 vaccine hesitancy, especially in Kenya and among females in both populations, where acceptance rates were lower. Expansion of the screening program to incorporate antibody (serology) tests, is also highly recommended in the present circumstance. Equitable distribution of vaccines globally should be encouraged and promoted to adequately cover low- and middle-income countries. To enhance effective combat on vaccination hesitancy and apprehension in different countries, mitigation techniques unique to those countries must be adopted.

## Introduction

Coronavirus disease 2019 (COVID-19) is a notably highly infectious disease caused by acute respiratory syndrome coronavirus 2 (SARS-CoV-2)^1^. The novel virus has spread rapidly putting to test every country’s governance and healthcare systems in a bid to curb and prevent further deaths and looming economic crises. Since the first official report in December 2019 from Wuhan, China, COVID-19 has spread to 221 countries and territories, infected 222,042,944 people, resulted in 4,590,307 deaths and fortunately 198, 669,095 recoveries as of 7th September 2021^2^.

The resurgence of the 4th and 5th waves of COVID-19 and the emergence of mutant strains further complicates the gains previously made in recent months worldwide. Increased agony generated by a spreading virus in a large population, can be quelled by developing effective and safe vaccines that are acceptable to the community. The uptake of vaccines that are certified and approved by internationally recognized regulatory bodies is therefore a game-changer towards overall combat of the veraciously and rapidly mutating SARS-CoV-2 virus^3^. However, acceptance rates for the COVID-19 vaccine vary widely around the world and hence become an impediment^4,5^. In this regard, an up-to-date comprehension of the beliefs that underlie COVID-19 vaccine hesitancy and the characteristics of persons who are less inclined to accept a vaccine or vaccination necessity, or mandate, is necessary for effective and comprehensive immunization strategies^6–9^. The effectiveness of vaccination campaigns to combat the coronavirus illness (COVID-19) depends on more than just vaccine efficacy and safety. The public’s and healthcare professionals’ acceptance of vaccines seem to be pivotal to the pandemic's successful control^7^.

A review conducted across African countries reported studies with low vaccine acceptance rates to a worrying rate of 6%, while 29.6% of the included cross-sectional studies, reported lower than 50% acceptance rates^10,11^. Misperceptions about the COVID-19 vaccine’s safety, effectiveness, hazards, and risks, as well as mistrust in the organizations in charge of vaccination programs, have been routed as significant contributing factors to hesitancy^4^. Other similar studies have also reported that fear and apprehension of vaccination are highly linked to skepticism of the pharmaceutical sector, findings from clinical trials, ineffective vaccination advertising with confusing information, false information from social media, and worries about getting sick or experiencing vaccine adverse effects^12–14^. To overcome vaccination hesitancy and apprehension in parts of Africa, mitigation techniques unique to Africa must be adopted^15^, while benchmarking from other developed nations as uniquely initiated in our comparative study.

In Kenya, vaccination uptake is far lower than that observed in other countries, and it continues to be a serious problem that necessitates attention^15,16^. It is critical to understand the underlying reasons and factors for the hesitancy and apprehension of individuals from uptaking these approved vaccines. It is against this complex background that this study aimed to determine the availability and acceptance rates of SARS-CoV-2 vaccines, among Kenyan and Hungarian residing populations and the underlying reasons contributing to the hesitancy or apathy of its uptake. It is imperative that with sufficient information adduced through our findings, inform the relevant authorities from an epidemiological perspective.

## Methods

### Target population

The research study targeted participants currently residing in Kenya, an African country, and Hungary a European country. Kenya was identified as an important target population having adopted the COVID-19, WHO guidelines and protocols at an early stage of the pandemic, compared to most of the other African countries. However, there seemed to be a unique COVID-19 vaccine acceptance and hesitancy population in worrying proportions, therefore necessitating its inclusion in our comparative study. Hungary constituted an important target population due to its central geographical location and position in the European continent. Europe was reportedly identified as the leading continent in COVID-19 strict adherence to the laid down COVID-19 guidelines and protocols. It was therefore imperative and interesting to compare and understand the unique disparities in acceptance rates of these two representative populations. A survey through an online questionnaire containing a total of 25 well-structured questions regarding COVID-19 disease was administered from April to August 2021, to adult participants (18 years and above), who had voluntarily consented to participate in the study. Our research was approved by Kenyatta University Ethics Review Committee and duly granted an approval number, PKU/2451/E1582. The research was performed in strict compliance with the guidelines and regulations as stipulated by Kenyatta University's ethical standards and the EU GDPR rules and regulations. Informed consent was obtained from all participants in digital format through the launched and programmed questionnaire tool link.

### Sampling design

A non-probability, snowball sampling design was deemed appropriate for our study. A survey questionnaire tool link was expeditiously disseminated through online platforms such as social (Facebook, WeChat, WhatsApp, Twitter, and Instagram) and professional (LinkedIn, yahoo, and Gmail) networks. To reach a wider community, the designed questionnaire was also translated from English to Swahili (One of two Kenya’s national languages that function as lingua francas) and Hungarian (Hungary’s national language). All inquiries were anonymized to guarantee privacy. In addition, no unique personal identifier was requested for disclosure under the terms of the granted ethical approval, the current EU GDPR rules, and in adherence with the Helsinki declaration of ethical principles. For successful inclusion into the study, participants had to fill in at least 22 (90%) survey questions and be ≥ 18 years old. In addition, the target participants at the time of undertaking the survey had to be living in either of the two target countries. All questionnaire feedback responses that did not meet the inclusion criterion were excluded from the study and their associated metadata was removed from our Microsoft Excel sheet before performing any advanced statistical analysis. A complete case deletion analysis approach was used on questionnaire feedback with missing data that fell below the established inclusion criterion^17^. Our acceptable sample size was determined and we targeted a minimum of 385 participants from each country^18–20^. The calculation was set at a 95% confidence interval (CI) and a 5% margin of error (ME)^18,19^.

### Questionnaire validation

For questionnaire validation, the new English language-developed questionnaire and its subsequent Swahili and Hungarian-translated questionnaire items passed through preliminary pilot testing and rigorous revisions guided by clear and effective validation phases and techniques^21^. In principle, a pilot test among our intended participants for initial validation was launched and tested. The final version of the new questionnaire was administered to a large representative sample of respondents (80, Kenya and 40, Hungary) from both countries. A forward questionnaire translation strategy was adopted for our study, involving two bilingual native speakers from each of our two countries of interest^22^. However, the translated versions were tested on a smaller sample size (40, Kenya and 20, Hungary)^23^. There are no strict guidelines for the sample size required to verify a questionnaire due to the variety of questionnaire types being employed^21^. The reliability and validity of the questionnaire were considered as the consistency of the survey results as adopted by other authors^21^. To facilitate a wide coverage, items were written in languages familiar to our target population, simple and short to guarantee optimum feedback responses^24^.

### Data analysis

Demographic variables, COVID-19 awareness, possible exposure, reasons associated with hesitancy in taking up a vaccine, choice of a vaccine, information on efficacy, and availability of vaccines among other important variables were tested to explore their associations with vaccine acceptance rates between the two distinct countries. Statistical Package for the Social Sciences (SPSS version 22.0) was used to analyze data imported from a comprehensively generated and coded excel sheet. One-way ANOVA was used to compare means from the two distinct populations. In addition, the Chi-square test was also used to determine any significant relationship between variables. Descriptive statistics for all variables were analyzed. We expressed results as mean ± standard deviation, range and median, and numbers expressed in percentage (%). The level of statistical significance was set at *p* < 0.05 with a 95% confidence interval.

### Limitations of the study

Despite the growing popularity of questionnaire surveys, perhaps for being an inexpensive, easy, and convenient means of data collection, our questionnaire survey had limitations too. Some rural and interior parts of the countries are not covered or connected to internet services and therefore the response rate was not high, to the expected margin earlier envisioned by the authors. Furthermore, while our questionnaires were strictly anonymous by design to guarantee participants' privacy, individual follow-up on social media platforms to optimize and increase response rate was also challenging. Finally, our survey only focussed on participants' willingness to uptake the available vaccines, that meet acceptable standards by the WHO, irrespective of their vaccination status.

### Ethical approval and consent to participate

Ethical clearance and approval were granted by Kenyatta University Ethics Review Committee (PKU/2451/E1582). In addition, informed consent was obtained after the nature and possible consequences of the studies had been fully explained to participants. All experiments were performed in compliance with relevant laws and institutional guidelines and under the ethical standards of the Declaration of Helsinki.

## Results

A total of 1960 participants successfully enrolled in the study had met the inclusion criteria, while 67 participants were excluded. The average age of the participants was 31.94, while the lowest and the highest age were 18 and 71 years respectively. The range was 53 and the SD was 9.315. Enrolled males were 988 (50.4%) while 972 (49.6%) were females. Of the respondents, 1528 (78.1%) were from Kenya while 432 (21.9%) were from Hungary. To justify the population disparity observed in our study between the two countries, Kenya was determined to have a population size of 47.5 million in 2019 by the Kenya National Bureau of Statistics (KNBS)^25^, while during the same period, Hungary was determined to have a population size of 9.7 million inhabitants according to the Hungarian Central Statistical Office (KSH)^26^. Regarding education, 45.5% were at the undergraduate level, 24.7% at the master’s level, 5.8% have attained secondary school level certification, 5.6% at Doctorate level and beyond and 3.6% of the respondents had attained tertiary level education. Of the respondents, 0.2% have primary school education while 0.2% did not attend school. In the comparison of means between gender and the highest level of education, without due regard to the country of origin, there was a striking significant difference*, p* = 0.007.

Concerning receiving any form of public awareness at the community level, regarding the importance of the newly developed vaccines against the SARS-CoV-2 virus associated with COVID-19 disease, our pooled analysis demonstrated that 63.7% of the participants affirmed (yes) while 36.3% affirmed negatively (no). To clearly illustrate the measurement, the survey question was: *Have you received any form of public awareness at your community level regarding the importance of the newly developed vaccines against the SARS-CoV-2 virus associated with COVID-19 disease*?. There was, however, no significant difference in public awareness between the Kenyan-residing participants and the Hungarian-residing participants, *p* = 0.300. Nonetheless, a small disparity in our segregated analysis showed that the Kenyan sample was rather informed/aware but in non-significant proportions, as demonstrated in the test of significance, than their Hungarian counterparts: 37.6% and 32.0% respectively. Sixty-two percent (62.4%) affirmed to be willing and ready to take up an approved vaccine against COVID-19 disease, while 37.3% affirmed negatively. Comparatively, there was a significant difference (*p* = 0.014) between the Kenyan and Hungarian-residing respondents. With regards to gender and vaccine acceptance rates in our pooled analysis, there was a notable significant difference between males and females, *p* = 0.001. The means for males and females were 0.29 and 0.46 respectively. Acceptance rates were higher in males than in females. Comparatively, vaccine acceptance rates were higher in Hungary than in Kenya; mean = 0.27, SD = 0.446, S. E = 0.045, and mean = 0.40, SD = 0.492, S. E = 0.026, for the Hungarian and the Kenyan population samples respectively. Reasons given for their negative responses were as follows: Belief (5.1%), culture (0.5%), religion (2%), the efficacy of the vaccine (26%), the safety of the vaccine (59.2%), and accuracy of information about the developed vaccines (54.1%). Multiple choice selection was enabled for this question.

**Figure 1:**
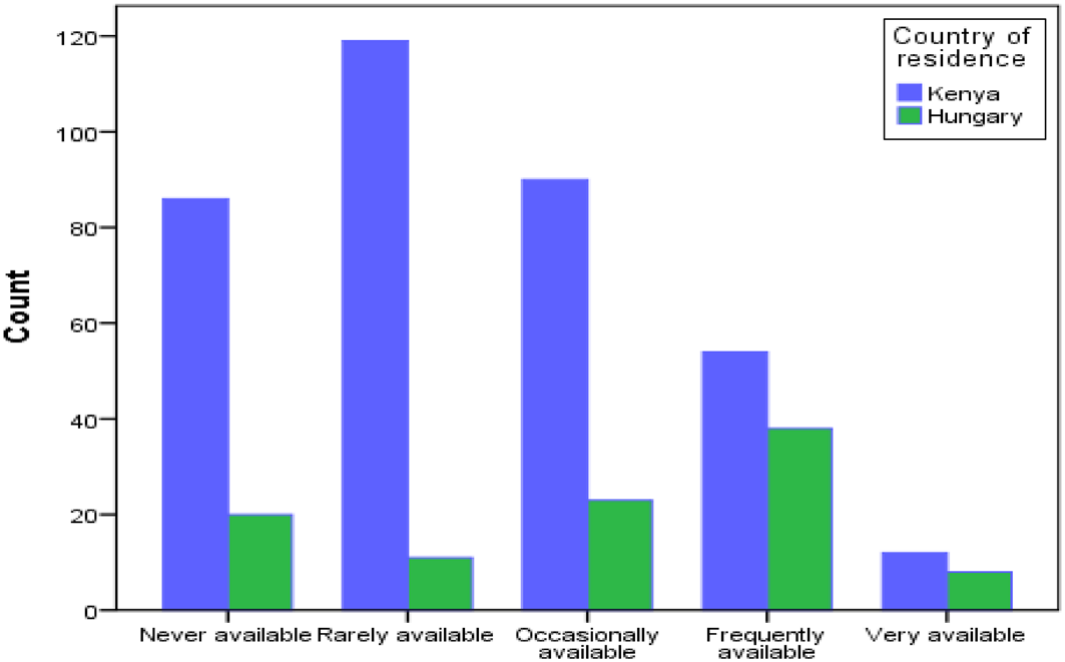
The figure represents a comparative display of the rate at which vaccines are perceived as available between the two sampled populations in Kenya and Hungary (1–5 Likert scale, the x-axis represents responses from respondents, the y-axis represents the count in frequency).

Regarding the availability of internationally approved biological vaccines against COVID-19 disease in the respondents’ countries of residence, a categorized Likert scale was developed, and the results analyzed as follows: Never available (23.2%), rarely available (28.1%), occasionally available (24.5%), frequently available (19.9%) and very available (4.3%). Figure 1, visually represents the segregated comparative results. There was, however, a significant difference between responses from Kenya and responses from Hungary, *p* = 0.001, Table 1.

**Table 1:** A table showing that there was a significant difference between responses from the two different countries studied, *p* = 0.001.

	Value	Degree of freedom (df)	Sig. (2-sided)
Pearson Chi-Square	38.792a	4	.000
Likelihood Ratio	38.289	4	.000
Linear-by-Linear Association	21.969	1	.000
N of Valid Cases	1960.0000		

**Figure 2:**
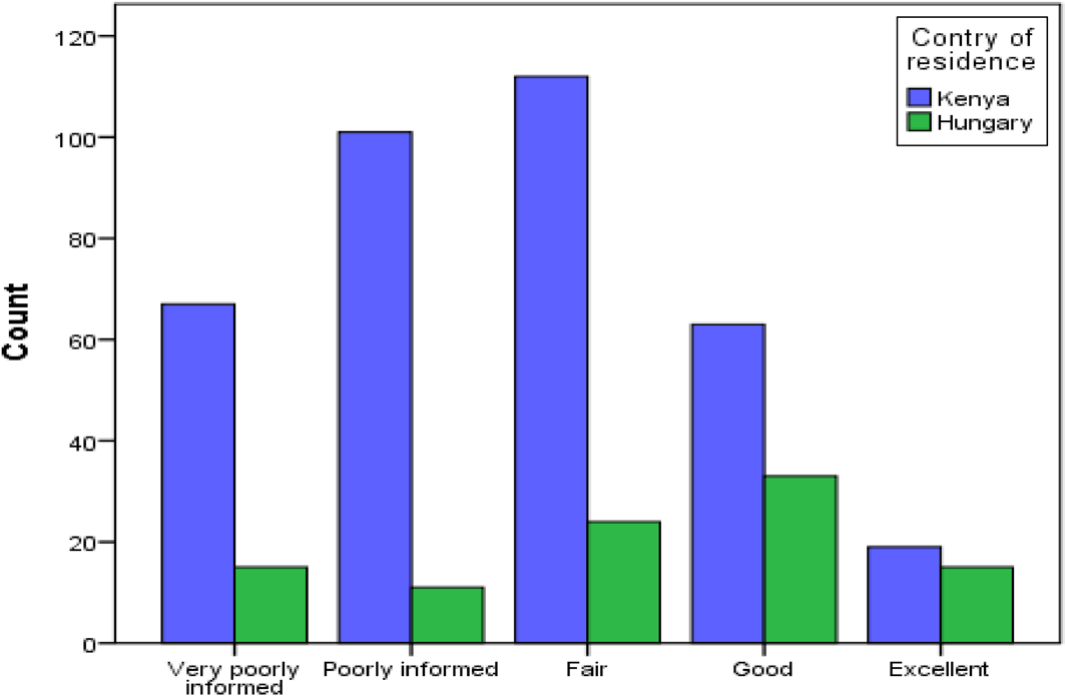
The figure demonstrates the significant difference between the two sampled populations regarding knowledge of vaccine efficacy, (1–5 Likert scale, the x-axis represents responses from respondents, the y-axis represents the count in frequency).

As to whether respondents had sufficient information and knowledge on the efficacy of the already developed vaccines against the SARS-CoV-2 virus, their responses were obtained on a defined Likert scale as very poorly informed (18%), poorly informed (24.3%), fair (29.5%), good (20.8%), excellent (7.4%). The results are summarised in the graph below (Fig. 2) with clear disparities demonstrated. There was a significant difference in the level of knowledge on vaccine efficacy between the two populations, *p* = 0.001, Table 2.

**Table 2:** The table shows a statistical output on variation in information/knowledge acquired on the efficacy of the developed vaccines against the SARS-CoV-2 virus.

Country	Descriptives and the output significant variation								
	N	Mean	Std. Deviation	Std. Error	95% Confidence Interval for Mean		Minimum	Maximum	Sig
					Lower Bound	Upper Bound			
Kenya	1528 (78.1%)	1.63	1.127	.059	1.51	1.75	0	4	0.000
Hungary	432 (21.9%)	2.22	1.281	.129	1.97	2.48	0	4	
Total	1960	1.76	1.185	.055	1.65	1.87	0	4	
df	1								

On inquiry about the advantage of getting immunized against the SARS-CoV-2 virus in the fight against the global COVID-19 pandemic, 24.7% said it was not necessary, slightly necessary (13%), moderately necessary (26.4%), very necessary (25.5%) and extremely necessary (10.4%). Comparatively, there was not any significant difference between responses from the Kenyan-residing participants and the Hungarian-residing participants, *p* = 0.891.

Without a laboratory diagnosis, 27.5% of respondents perceived to have been exposed to the SARS-CoV-2 virus, by developing three or more commonly known symptoms associated with COVID-19 disease. Of the respondents, 35.7% perceived not to have been exposed while 36.8% were not sure but with a probability of exposure perception (maybe). With regards to symptoms presentation, the majority (61.2%) of the respondents stated they had experienced headache, 48.3% of the respondents claimed to have presented with cough, fever (37.1%), loss of smell (anosmia) 21.8%, complete loss of taste (ageusia) or partial loss of taste (hypogeusia) 18.4%, breathing difficulties (dyspnoea) 14.6%, fatigue 57.5%, sore throat 49.7%, and finally diarrhea 15%. Through a laboratory diagnosis, few (9.7%) respondents reported having been exposed (contracted) to the SARS-CoV-2 virus, while most (80.3%) of them were not exposed to the virus. By statistical comparison, there was not any significant difference between the two countries, *p* = 0.504.

**Figure 3:**
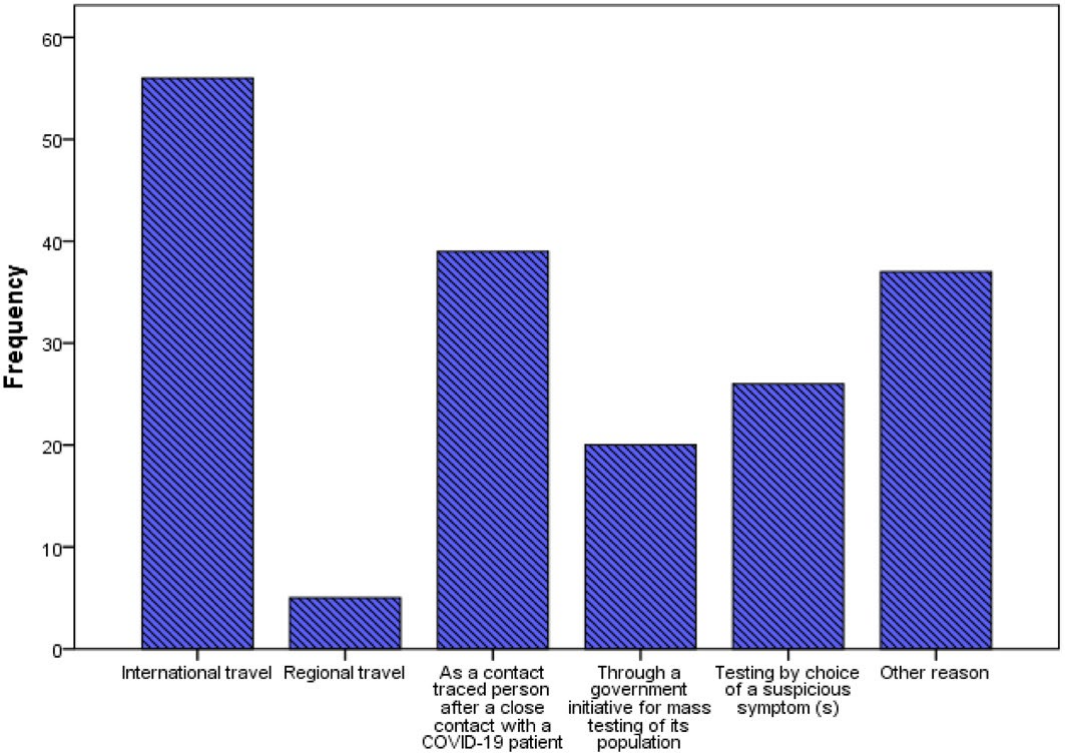
This figure represents an analysis of the reasons cited by participants for being screened for COVID-19 disease by participants.

In response to getting screened for COVID-19, 37.6% of the respondents affirmed (yes) while 62.4% of the respondents affirmed negatively (no). Given their comparative nature, there was a significant difference between responses from the Kenyan-residing respondents and the Hungarian-residing respondents, *p* = 0.001. The reasons stated for being screened were for international travel (30.6%), regional travel (2.7%), as a contact traced person after close contact with a COVID-19 patient (21.3%), through a government initiative for mass testing of its population (10.9%), testing by choice of a suspicious symptom(s) (14.2%), other reason (20.2%). Figure 3 summarizes the reasons given by the respondents. There was a significant difference (*p* = 0.001) between the reasons given for getting screened among responses from the two different countries sampled.

With regards to the choice of vaccine, the majority (46.1%) preferred internationally developed and approved vaccines, while 13.4% preferred locally developed vaccines. However, 40.5% were okay with either the source of the vaccine. There was statistically no significant difference (*p* = 0.324) between responses from the two different populations sampled. Concerning acceptance of being screened for COVID-19 disease, using antibody (serology) tests, the majority (62.7%) of the respondents affirmed that they could take the test, 29.6% were not sure but had a probability of acceptance, while only 7.6% of the respondents claimed they could not take the test. Regarding acceptance of screening by using antibody tests, there was no difference (*p* = 0.614) between responses obtained from the Hungarian-residing participants and those of the Kenyan-residing participants.

## Discussion

Despite global calls for the adoption of mass screening strategies and immunization of the population at risk, other countries refused to adopt and implement the laid down measures. Meanwhile, the SARS-CoV-2 virus was rapidly mutating and spreading rapidly across many countries and territories^2^. Perceptions and speculations regarding the whole idea of the safety of vaccines, the accuracy of vaccines, the efficacy of vaccines, public awareness, screening programs, and rolled-out immunization programs were all received with mixed reactions from different quarters. While weighing the importance and harmful consequences of vaccination, people appear to accept a higher risk of catching an infection as opposed to agonizing from vaccine side effects^5^. This comparative study creates a better understanding of the most probable intervention strategies applicable to different populations. It further opens an avenue of discussion, to build confidence among the hesitant and apprehensive population on approved vaccines by the WHO. Tremendous strides have been made in manufacturing effective and safe vaccines against the SARS-CoV-2 virus^3^.

In our segregated analysis, 37.6% of the Kenyan respondents had received public awareness compared to 32.0% of their Hungarian counterparts. Public awareness at the community level is critically a paramount exercise, to constructively build and sustain confidence among target participants of any immunization program, whether in developed countries or developing countries. Due to strictly instituted lockdown control measures, there seems to have been low outreach and campaign programs in both countries. Immunization programs are desirable if executed promptly and with requisite acceptance and coverage^27^. A multidisciplinary and multisectoral approach should be adopted to enhance the wide coverage of information dissemination regarding SARS-CoV-2 vaccines. Such an exercise will allay in-depth fear, resistance, panic, doubt, suspicion, and anger among people^27^. Of the respondents, 37.2% claimed that they were not ready to take up the vaccine. These findings were slightly higher (62.4%) than those of a similar study in Ethiopia (40.8%)^28^. However, very low acceptance rates were observed in Israel: males, 27.3% among Jews, and 23.1% among Arabs, while among females, the figures were much lower: 13.6% among the Jews and 12.0% among the Arabs^29^. The study observed that the vaccine uptake was significantly lower among the Arabs compared with the Jewish participants^29^. A separate study reported that countries with low vaccine acceptance rates were Kuwait (23.6%), Jordan (28.4%), Italy (53.7), Russia (54.9%), Poland (56.3%), the United States (56.9%), and France (58.9%)^6^. In addition, another systematic review sampling 8 different countries reported that the highest and lowest hesitation were observed in France (47.3%) and Brazil, respectively (9.6%). The study further unveiled that women, those under the age of 29, residents of rural areas, and people with lower reported incomes tended to be more apprehensive of vaccine uptake^9^. There have been reports of low acceptance rates for the COVID-19 vaccination in the Middle East, Russia, Africa, and several European nations. This could pose a significant obstacle to efforts being made around the world to contain the epidemic^6^. In contrast, in other systematic reviews, a wide range of COVID-19 vaccine uptake rates has been reported^7,30^. First, in East and Southeast Asia, there was a rather high level of acceptance. This includes acceptance rates of more than 90% in China, Malaysia, and one research from Indonesia. More than 80% of the general population in two more studies conducted in China and a further 79.8% of respondents in a survey conducted in South Korea were said to be in favour of vaccination^7,30,31^. A different survey reported that countries with the highest acceptance rates of the COVID-19 vaccine, among adults who represent the general population were Ecuador (97.0%), Malaysia (94.3%), Indonesia (93.3%), and China (91.3%)^6^.

It is important to note from these findings, that public awareness directly corresponds to vaccine uptake among respondents in our study survey. It is therefore imperative, to address these aspects with urgency. Comparatively, more, and robust sensitization was needful in Kenya (Africa) compared to Hungary (Europe) where acceptance rates were significantly high according to our test of significance. The highest factors of concern were based on the safety of vaccines (59.2%), the accuracy of information about the developed vaccines (54.1%), and the efficacy of the vaccines (26%). Furthermore, a larger proportion of the respondents (71.8% cumulatively) claimed to have insufficient information regarding the efficacy of vaccines being rolled out in their countries. Being a multisectoral screening and immunization campaign, knowledgeable health professionals in various cadres should be facilitated to educate the wider community within their reach. Factors influencing COVID-19 vaccine acceptance are as critical as the discovery and development of the vaccine^32^.

Compared to the Hungarian residing population, the availability of vaccines among the Kenyan residing population was perceived to be very low and, in some cases, never available at all. The procurement of safe and efficacious vaccines acceptable to the general population in sufficient amounts should be strengthened by the national governments if the war against the COVID-19 disease pandemic must be won. The incorporation of emergency response COVID-19 drills at the community level should be adopted too. It has been cited that Israel adopted a robust vaccination rollout strategy that has been largely termed successful^33^.

A large percentage (64.1% cumulatively) of the respondents were not sufficiently convinced that the uptake of vaccines against COVID-19 disease was very important. A similar study conducted in the United Arab Emirates (UAE) reported that to acquire herd immunity against COVID-19, a high percentage of the population must be vaccinated, and to attain this, the immunization campaigns should focus on certain expectations and motivating factors, regarding each target group to fully conquer the challenge of vaccination hesitancy^34^. A separate study cited that high vaccine uptake and acceptance rates are needed for COVID-19 immunization programs to be successful and acquire herd immunity targets and objectives^35^. Without screening for COVID-19 disease, a majority (64.3% cumulatively) of the respondents perceived to have been exposed to the SARS-CoV-2 virus by exhibiting 3 or more symptoms closely associated with its infection. On the flip side, 62.4% of the respondents had not been screened for the disease. This complicates the war against the pandemic as most individuals feel assured to have developed their natural immunity without certainty of prior SARS-CoV-2 viral infection. Adoption of antibody (serology) screening can be included as an alternative screening strategy. The presence of the immune antibodies directly corresponds to prior SARS-CoV-2 viral infection. These antibodies when detected, show the body’s capability to fight off the specific viral infection. The Centre for Disease Control and Prevention (CDC) recently availed interim guidelines on how laboratories, public health staff, and healthcare service providers should use antibody tests^36^. An overwhelming 92.4% of the respondents, claimed they were certain or somehow could go for antibody screening tests if rolled out. Only 7.6% claimed they could not take the serological tests. This is a very high acceptance rate for the test that should be adopted overly. In addition, it is a sure measure to ascertain the level of herd immunity attained in a specific population^34^. Most of the respondents went for screening not because they desired to, but because it was the only way they could be allowed to travel beyond their countries’ territories or because of being tracked down, on account of suspicion of close contact with an infected subject. Furthermore, only 10.9% of the respondents had been screened through their government’s initiative for mass testing. Exposing the dire need for a robust and intentional screening program that is equally safe and acceptable to a wider community. Alternative screening methods should therefore be encouraged and promoted to guarantee a wide coverage of mass screening. Sufficient and adequate coverage of screening programs averts the need for unnecessary lengthy lockdowns and restrictions, due to their long-term social-economic impacts. Movement restrictions are not a feasible long-term plan and they have detrimental socioeconomic effects^37^.

As different countries are rushing to domesticate vaccine production in their own countries, it is important to take note that from our study, a majority (46.1%) of the respondents preferred internationally developed vaccines as opposed to those who preferred locally produced vaccines (13.4%). It is therefore critical, that the National government expand its training programs in building enough trust among its citizens, to promptly accept their locally developed vaccines. Some developing countries are challenged with existing and intrinsic broken-down healthcare systems, that may not be trusted by their citizens with such delicate and human-sensitive vaccine productions. Contrary to our study and before vaccines were rolled out globally, a study done in Turkey demonstrated high acceptance rates for locally developed vaccines^38^. Surveillance and monitoring in the general population are paramount at every stage during the vaccination rollout^39^. A separate study identified that implementation of the health belief model to captivate communities can promote the demand, uptake, and equitable distribution of vaccines thereby reducing the probability of vaccine hesitancy^40,41^.

## Conclusion

Public perceptions and unsubstantiated claims on the premise of vaccine safety, the accuracy of information relayed, the efficacy of vaccines, public awareness, screening programs, and implemented immunization programs were all met with varying degrees of response in the two countries. The fight against COVID-19 disease will be won through concerted efforts by governments working closely with the community. Dissemination of accurate and valid information is paramount to allay any form of hesitancy, fear, apathy, discontent, or varying levels of dissatisfaction among people. Concerns regarding the safety of vaccines, the accuracy of information about the developed vaccines, and the efficacy of the vaccines, are significant factors that must not be overlooked but promptly addressed by the authorized health regulatory bodies, especially in Kenya, where acceptance rates were lower. This will encourage communal participation and uptake of approved vaccines rolled out in different countries, meeting sufficient acceptance rates. In addition, equitable distribution of vaccines globally should be encouraged. To enhance effective combat on vaccination hesitancy and apprehension in different countries, mitigation techniques unique to those countries must be adopted. Finally, more unique comparative studies of this nature should be considered to improve epidemiological approaches modeled for specific countries, to realize better outcomes and far-reaching benefits.

## Data Availability

Data are available from the corresponding author upon reasonable request.
